# 

**Published:** 1869-03

**Authors:** 


### AMERICAN DENTAL ASSOCIATION.

The next annual meeting of the American Dental Association, is one to which our whole profession look with interest, and as one of great importance.

It will be productive of good results in proportion as all of the members and delegates go up to its meeting with a just sense of the responsibility resting upon them, every one prepared to accomplish well the work devolving upon him. Let every one have something put in proper form, so that if occasion calls for it, or an opportunity is offered, it may be presented, so as to bless others. But this does not constitute all the preparation, let every one examine himself, and ascertain in what respect his deficiencies exist, and then let him be on the look-out for that which will strengthen his weakness, and for that which will enlighten that which is dark—for knowledge to supplant ignorance. We are never so well prepared to learn, as when fully aware of our lack of knowledge.

In addition to the general work of preparation by all of the members of the Association, there is a portion of the membership constituting various committees, whose duty it is to make particular preparation upon special subjects, every one of which, we trust, will be fully prepared. We would suggest that the members composing each committee confer together, and let their reports be the result of wise and deliberate counsel.

We append the names and addresses of the persons composing the various committees :

*Committee of Arrangements*—J. G. Ambler, New York; L. W. Rogers, Utica, N. Y.; L. S. Straw, Newburg, N. Y.

*Pathology*—W. H. Atkinson, New York; J. S. Latimer, New York; Moses DeCamp, Mansfield, O.; Geo. S. Moffatt, Boston; A. B. Robbins, Meadville, Pa.;

*Physiology*—W. H. Morgan, Nashville, Tenn; A. Westcott, Syracuse, N. Y.; H. S. Chase, St. Louis, Mo.

*Chemistry*—T. L. Buckingham, Philadelphia, Pa.; H. A. Smith, Cincinnati, O.; A. Lawrence, Lowell, Mass.

*Education*—J. Taft, Cincinnati, O.; F. J. S. Gorgas, Baltimore, Md.; M. S. Dean, Chicago, Ill.

*Dental Literature*—R. W. Browne, New London; L. D. Shepard, Boston; J. McManus, Hartford, Ct.

*Histology and Microscopy*—J. H. McQuillen, Philadelphia, Pa.; W. W. Allport, Chicago, Ill.; J. S. Dodge, Jr., New York.

*Operative Dentistry*—C. R. Butler, Cleveland, O.; J. S. Knapp, New Orleans, La.; W. H. Allen, New York.

*Mechanical Dentistry*—John Allen, New York; N. W. Kingsley, New York; C. H. Harroun, Toledo, O.; J. A. McClelland, Louisville, Ky.; S. B. Palmer, Syracuse, N. Y.

*Prize Essays*—G. H. Cushing, Chicago, Ill.; A. L. Northrup, New York; W. F. Morrill, New Albany, N.Y.; E. C. Francis, New York; F. N. Seabury, Providence, R. I.

*Voluntary Essays*—M. S. Dean, Chicago, Ill.; A. M. Moore, Lafayette, Ind.; W. P. Horton, Cleveland, O.

*Dental Therapeutics*—E. A. Bogue, New York; I. J. Weatherbee, Boston; A. P. Morrill, New York.

*Publication*—Homer Judd, St. Louis; Edgar Park, St. Louis; Wm. N. Morrison, St. Louis.

*Surgical Instruments and Appliances*—W. C. Horne, New York; Corydon Palmer, Ohio; J. A. Bishop, New York.

### The Dental Register

### OF THE WEST.

*Issued Monthly, at $3 a Year in Advance.*

DEVOTED TO THE INTERESTS OF THE DENTAL PROFESSION.

EDITED BY J. TAFT AND G. WATT.

The volume commences in January and closes in December.

The Register being issued monthly is always fresh, therefore indispensable to the practitioner who desires to keep posted up with the new inventions and improvements which are daily developed. Neither expense nor effort will be spared to give value and variety to its contents. Articles will be illustrated with well executed engravings whenever they will add to the interest or assist in explanation.

Its extensive circulation and frequency of issue makes it one of the BEST DENTAL ADVERTISING MEDIUMS in the United States.

RATES OF ADVERTISING.

One page, 1 year, $50. Half page, 1 year, $30. Quarter page, or card, 1 year $20.

All advertising bills payable in advance, or at furthest after the issue of the first number containing the advertisement. All transient advertisements to be paid for in advance

☞ CONTRIBUTIONS SOLICITED.

Address, J. TAFT, or "DENTAL REGISTER," Cincinnati Ohio.

Business Communications to

**J. TAFT, Publisher,**
No. 117 West Fourth St.

